# Host–Guest Complexes HP-β-CD/Citrus Antioxidants: Exploratory Evaluations of Enhanced Properties in Biodegradable Film Packaging

**DOI:** 10.3390/antiox12030763

**Published:** 2023-03-21

**Authors:** Giovanni Gallo, Domenico Zannini, Barbara Immirzi, Alessandra De Bruno, Gabriella Fiorentino, Giovanni Dal Poggetto

**Affiliations:** 1 Department of Biology, University of Naples Federico II, Complesso Universitario di Monte Sant’Angelo, 80126 Napoli, Italy; 2 Division of Microbiology, Faculty of Biology, Ludwig-Maximilians-Universität München, 82152 Martinsried, Germany; 3 Institute of Polymers, Composites and Biomaterials, National Research Council, Via Campi Flegrei 34, 80078 Pozzuoli, Italy; 4 Department of Chemical Sciences, University of Naples Federico II, Complesso Universitario di Monte Sant’Angelo, 80126 Naples, Italy; 5 Department of AGRARIA, University Mediterranea of Reggio Calabria, 89124 Reggio Calabria, Italy

**Keywords:** pectin, antioxidant, citrus waste, circular economy, cyclodextrin

## Abstract

The aim of this work was to exploit the antioxidant potential of molecules recovered during the pectin purification process of *citrus lemon* waste and to encapsulate them in stable pectin films, with a view to a green and circular economy process. Antioxidant molecules were recovered during the pectin purification process, further recovering matter from the waste. Seven molecules were identified and quantified, and the antioxidant power of the mixture and its stability over time was evaluated. To improve the stability of the bioactive fraction, this was complexed with 2-hydroxypropyl-β-cyclodextrin (HP-β-CD); indeed, this procedure increased their thermal stability from 120 °C up to 250 °C, as verified by thermogravimetry. Furthermore, the most promising complexes were studied under autoclave-like conditions (120 °C, 28 min) to simulate thermal sterilization. The antioxidants and HP-β-CD were combined in a pectin film, showing increased stability over time (up to three times) compared to uncomplexed antioxidants. This process represents a first step towards the development of applicable devices for the delivery of antioxidant molecules.

## 1. Introduction

The agri-food industry generates important sub-products that mostly end up in waste dumps or are incinerated. The reuse of these by-products can bring new revenue streams and solutions to environmental and social challenges. Among the waste from the agri-food chain, a large part is made up of citrus fruits, which are consumed all over the world. Between 1971 and 2020, citrus production increased significantly from 42.1 million to 158 million tons in 2020. The processing of citrus is very inefficient, as the waste produced reaches about 40–60% of the original mass. Therefore, it is extremely important to recycle it [1]. The most common wastes from citrus processing consist of peels, seeds, and fibrous residues, which are generally rich in antioxidants, cellulose, hemicellulose, and pectin [1].

Pectin is a biodegradable heteropolysaccharide composed mostly of galacturonic acid units linked by α(1–4)-glycosidic bonds; along the polygalacturonic acid chain, rhamnose residues are inserted at random positions [2]; however, composition and structure of pectin is very complex and depends on several factors: extraction method, source material, storage, and maturity of the source.

Antioxidants, on the other hand, are part of the essential oils in citrus waste. This includes flavonoids, alkaloids, coumarins, limonoids, carotenoids, phenolic acids, and many polymethoxylated flavones [3,4], which are utilized for their properties (antioxidant activities, flavor, in food as well as the cosmetic industries [3,5,6,7].

Pectin and antioxidants can be combined with cyclodextrins (CD) to create smart materials with improved properties driven by growing consumer interest in health, nutrition, food safety, and environmental issues. These materials can serve as biodegradable packaging, thereby reducing the use of plastics. These last are an increasing environmental burden as only less than 5% are recycled, leading to rapid accumulation in the environment [8]. This research is moving toward the use of biopolymers as an alternative.

In this regard, CDs are especially promising as they resemble a three-dimensional structure comparable to hollow truncated cones and with different polarities: hydrophilic rims and hydrophobic cavities. From a molecular point of view, CDs are composed of α(1,4)-linked cyclic oligosaccharides, and natural CDs include at least six (α-CD), seven (β-CD), or eight (γ-CD) glucose units.

Due to this particular shape and properties, CDs have the possibility to entrap molecules in their cavity, forming the so-called host–guest complexes. These are supramolecular structures stabilized via noncovalent bonds (van der Waals, hydrogen bonds, and hydrophobic interactions, etc.).

**Figure 1:**
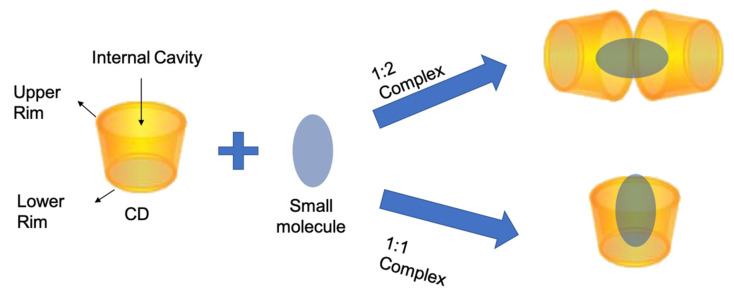
The schematization of CDs most common complexes.

Different complexes can be formed (Figure 1) depending on the host–guest molar ratio [9]. These kinds of complexes can improve the solubility of poorly soluble molecules, protect them from oxygen, heat and UV degradation and achieve a controlled release over time [10,11].

Beyond natural CDs, there is a wide variety of modified versions; as an example, 2-Hydroxypropyl-β-cyclodextrin (HP-β-CD) is a derivative version of β-CD characterized by improved water solubility and lower toxicity [12]. Thanks to these features, HP-β-CD is widely used in the pharmaceutical and food industry (nanoparticles, protecting agents, smart packaging, etc.) [10].

In this work, we characterize the antioxidant molecules obtained from the extraction of pectin from *citrus lemon* waste and set up a procedure for their encapsulation in HP-β-CD in different molar ratios, up to theoretical 1:2 (antioxidant/HP-β-CD) [13]. First, we evaluated and confirm complexes formation through FTIR analysis; subsequently, the resultant complexes were apprised of their thermal properties, and the most stable heat samples were subjected to autoclave-like conditions. Then, we dispersed the complexes in a pectin matrix and evaluated them for their antioxidant activity over time.

Collectively, our approach allows the recycling of citrus waste components and, at the same time, lays the foundation to utilize them in bioactive films. This, in turn, could help solve the growing problem of non-degradable plastic packaging and, at the same time, improve the food shelf life.

## 2. Materials and Methods

### 2.1. Material and Chemicals

*Citrus lemon* waste was kindly supplied by Università Mediterranea of Reggio Calabria (Italy), with a moisture content of about 15% by weight. Glacial acetic acid (HAc), isopropanol, ethanol, acetone, 2-Hydroxypropyl-β-cyclodextrin (HP-β-CD), Folin–Ciocalteu (F–C) reagent, gallic acid, and 2,2-diphenyl-1-picrylhydrazyl (DPPH) are all analytical grades purchased by Sigma-Aldrich (Darmstadt, Germany).

### 2.2. Extraction of Pectin and Bioactive Molecules Fraction from Citrus Lemon Waste

The procedure adopted to extract pectin was described by Zannini et al. [14]. Briefly, 20 g of powder citrus waste were dispersed in 200 mL of a solution of 3% HAc (*v*/*v*) (pH = 2.6). The pectin extraction was performed at 90–100 °C for 6 h under a magnetic stirring. Afterward, the crude product was cooled to room temperature and filtered to separate the insoluble fraction from the liquid. Subsequently, the liquid fraction was centrifuged at 10,000 rpm for 40–50 min at 4 °C. The supernatant was added to two volumes of isopropanol, and precipitated pectin was filtered on a paper filter. Afterward, pectin was washed with ethanol and put in a vacuum oven at 60 °C overnight. The percentage yield of the pectin was determined through the following equation:

(1) $$Pectine\;yield\;\%=\frac{a}{b}*100$$

where *a* corresponds to the mass of pectin obtained, and *b* corresponds to the initial mass of citrus waste powder. The pectin collected was around 24%, and extraction was performed in duplicate. After pectin extraction, the remaining liquid fraction was evaporated using a rotavapor to remove the solvent (consisting of propanol and ethanol) and left under vacuum at 60 °C for 16–18 h. The recovered dry solid amount from 10 mL of liquid fraction was 164.6 mg. The antioxidant activity in this fraction was determined by resuspending 1 mg of powder in 1 mL of 50 mM Na-citrate at pH 4.8. Once resuspended, the total phenolic content and the antioxidant activity were assessed using the Folin–Ciocalteu method and the inhibition of DPPH, respectively (see Section 2.7.1 and Section 2.7.2).

### 2.3. Identification and Quantification of Antioxidant Compounds

In order to ascertain whether the remaining liquid fraction contained antioxidant compounds and to separate, identify and quantify them, the protocol described by Romeo et al. was followed with some modifications [15]. The chromatographic system was a UHPLC PLATINblue (Knauer, Berlin, Germany) provided with a binary pump system, Knauer blue orchid C18 column (1.8 µm, 100 × 2 mm) coupled with a PDA–1 (Photo Diode Array Detector) PLATINblue (Knauer, Berlin, Germany) and Clarity 6.2 software (London, UK). Extracts were filtered through a 0.22 μm nylon syringe filter (diameter 13 mm), and then 5 μL were injected into the system. The mobile phases used were: (A) water acidified with acetic acid (pH 3.10) and (B) acetonitrile; the gradient elution program consisted in 0–3 min, 5% B; 3–15 min, 5%–40% B; 15–15.5 min, 40%–100% B. Ultimately, restoration of the initial conditions was reached during analysis maintaining the column at 30 °C. The chromatographic method was validated through the injection of external standards at several concentrations (between 1 and 100 mg/kg) and related calculation of regression equations, correlation coefficient (R^2^), limits of detection (LOD) and quantification (LOQ) for each standard was reported in Table 1. The results were expressed as mg/kg of citrus by-products dry weight.

### 2.4. Preparation of the Complexes HP-β-CD with an Antioxidant Fraction

HP-β-CD complexes with the recovered fraction containing antioxidants were obtained by the following procedure: different amounts of HP-β-CD (Table 2) were added to a constant volume of the bioactive fraction (100 mg of dry mass in 6.09 mL of water). The mixture solutions were kept under magnetic stirring for 48 h at room temperature to facilitate the formation of the HP-β-CD inclusion complexes denoted by the appearance of turbidity [16]. After this time, the solvent was removed under vacuum until constant weight, and the sample was analyzed through different techniques. Different inclusion complexes were prepared varying the molar ratio of antioxidant/HP-β-CD fixing a theoretical 100% at a molar ratio of 1:2, considering for the antioxidant fraction a medium molecular weight of 590 g/mol (based on molecules found in citrus peel; see Section 3.2). Sample identification codes are reported in Table 2 [17].

### 2.5. Thermogravimetric Analysis 

Thermogravimetric analysis (TGA) of HP-β-CD, antioxidant fraction, and antioxidant/HP-β-CD inclusion complexes were performed using the thermogravimetric analyzer TGA/DTA Perkin-Elmer PyrisDiamond (Milan, Italy), equipped with a gas station. In general, a mass of 3–4 mg of each sample was placed in an open ceramic crucible and heated from 25 °C up to 900 °C at 10 °C/min, under a nitrogen flux of 30 mL/min. Moreover, after this preliminary screening, samples showing enhanced thermal stability were subjected to isothermal treatment: from 25 °C up to 120 °C at 10 °C/min, under a nitrogen flux of 30 mL/min and kept at 120 °C for 30 min, in order to mimic autoclave sterilization. All measurements were performed in duplicate.

### 2.6. Fourier-Transform Infrared Spectroscopy

Fourier transform infrared (FTIR) was employed to analyze samples in the powdered state in order to identify functional groups and host–guest complexes formation. Spectra were obtained using a PerkinElmer spectrometer (Paragon 500, Milan, Italy), equipped with a ZnSe attenuated total reflectance (ATR) crystal accessory. Spectra were acquired in the 4000–650 cm^−1^ range, at a resolution of 4 cm^−1^ (16 scans).

### 2.7. Antioxidant Assays

#### 2.7.1. Folin–Ciocalteu (F–C) Assay

This method is based on the transfer of electrons in an alkaline medium from phenolic compounds to form a blue chromophore consisting of a phosphotungstic/phosphomolybdic complex in which the maximum absorption depends on the concentration of the phenolic compounds.

Total phenolic content was determined in a 96 multiwell plate using gallic acid as standard and following the Folin–Ciocalteu method [18]. The mixtures were prepared in a final volume of 180 µL adding in the sample wells 20 µL of sample and 20 µL of Folin–Ciocalteu working solution. The plates were kept in the dark for 10 min; then, 50 µL of sodium carbonate was added, and incubation was prolonged for another 20 min in the dark. The absorbance was measured at 760 nm in a microplate reader (BioTek, Waldbronn, Germany) and phenolic content was expressed as micrograms of gallic acid equivalent per liter (mg_GAE_/L) of the sample. The results reported are the average of three independent experiments made in triplicate.

#### 2.7.2. DPPH Assay

The α,α-Diphenyl-β-picrylhydrazyl radical (DPPH) method is a colorimetric assay based on electron transfer that produces a violet solution in ethanol. This free radical is reduced in the presence of an antioxidant molecule, resulting in a colorless solution. The assay was performed at 517 nm, as already described [18]. Gallic acid was used as the reference compound. DPPH· inhibition percentage was calculated using the following formula:

(2) $$\%\;inibition=\frac{A_{0}-A_{t}}{A_{0}}*100$$

where *A*_0_ and *A_t_* were the absorbance values of the control (DPPH solution, without extract) and of the extract, respectively. The reported results are the average of three independent experiments each made in duplicate.

### 2.8. Preparation of Pectin Films with Antioxidant/HP-β-CD Complexes and Antioxidant Activity Stability over Time

Since HP-β-CD60 and HP-β-CD80 inclusion complexes had a thermal behavior that suggested the stability of complexes, these two were used for further encapsulation within pectin films. Therefore, pectin films containing different amounts of HP-β-CD60 and HP-β-CD80 were prepared by the solvent evaporation method.

Briefly, pectin and complexes prior to film preparation were sterilized at 120 °C for 20 min. Then, a solution of pectin in water (4% *w*/*v*) was prepared and mixed with HP-β-CD60 (10–20–30% *w*/*w* compared to pectin) under magnetic stirring. Once homogeneity was reached, 20 µL were dropped in 96 multiwell plates, and the solvent was removed by natural evaporation. As a control, a sample containing only pectin and uncomplexed antioxidants (corresponding to the antioxidant amount present in the sample 20% *w*/*w*) was prepared. The same procedure was applied for films with HP-β-CD80. All the samples were prepared in duplicate, and the antioxidant activity was measured at intervals from 0 to 120 h.

## 3. Results and Discussion

### 3.1. Side Extraction of Antioxidant Molecules

After pectin extraction, the remaining liquid fraction was dried and analyzed for its polyphenol content using the F-C method and for its antioxidant activity against DPPH. The former method uses the redox reagent F-C to form a blue complex with phenols or other antioxidant compounds that can be quantified by measuring the absorbance at 760 nm [19]. On the other hand, the DPPH assay is a widely used and simple approach to assess the free radical removal activity of antioxidant compounds. The technique is associated with the removal of the free radical DPPH that interacts with a split electron to produce a strong absorbance at 517 nm. The results showed that the waste pectin fraction had an antioxidant compound content of 0.020 ± 0.005 mg_GAE_/L and a DPPH inhibition of 60 ± 6%.

### 3.2. Evaluation of the Antioxidant Molecules Extracted from Citrus Waste

Since the residual sample recovered from pectin extraction showed a strong antioxidant activity, an in-depth analysis of its composition was carried out through a UHPLC analysis.

**Figure 2:**
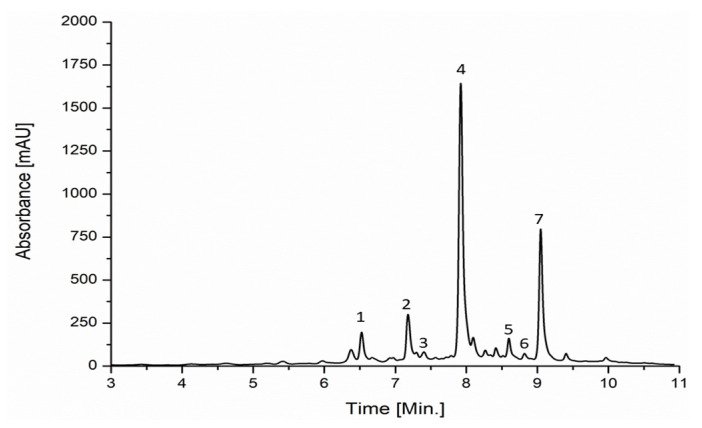
Example of chromatographic profile: peak designation was: (1) p-cumaric acid, (2) ferulic acid, (3) rutin, (4) eriocitrin, (5) narirutin, (6) naringin, (7) and hesperidin.

Table 3 reports the antioxidant molecules identified (mainly flavonoids) and their specific quantity (mg/kg). In particular, seven individual phenolic compounds were quantified on the analyzed extract, some of which were previously identified in a recent work carried out on citrus lemon by-products [20]. An example of the chromatographic profile is shown in Figure 2.

As presented, the main antioxidant component obtained as a by-product of pectin purification is eriocitrin, which consists of eriodictyol substituted by a 6-*O*-(alpha-l-rhamnopyranosyl)-beta-d-glucopyranosyl moiety at position 7 via a glycosidic bond [21]. Eriocitrin is the most common flavone in citrus fruits and is often used as a natural supplement because it has interesting biological activities, such as the ability to reduce lipid levels in the liver [22]. The second most abundant antioxidant is hesperidin, another flavanone glycoside, which has long been studied for its possible effects on human health [7]; recent studies have shown that it provides significant antioxidant, anti-inflammatory and neuroprotective effects in central nervous system disorders [23].

Finally, naringin and narirutin have also been found, albeit in smaller quantities. Naringin is a disaccharide derivative consisting of (*S*)-naringenin substituted with a 2-*O*-(alpha-l-rhamnopyranosyl)-beta-d-glucopyranosyl moiety at position 7 via a glycosidic bond. It has potent antioxidant activity and is an antineoplastic and anti-inflammatory agent [24]. Narirutin is a disaccharide derivative that is (*S*)-naringenin substituted by a 6-*O*-(6-deoxy-alpha-l-mannopyranosyl)-beta-d-glucopyranosyl moiety at position 7 via a glycosidic bond with a vasodilator effect; indeed, it has been shown to induce the release of nitric oxide and the activation of potassium channels in vascular muscle cells in rat mesenteric arteries [25]. The other extracted components are hesperidin, rutin, and p-cumaric acid; these molecules are still being studied with regard to their effects on human health; for example, their use is being tested as a supplement in people suffering from venous insufficiency [26].

More in general, flavonoid compounds have multiple health benefits as nutraceuticals and find application in food and herbal supplements [27].

### 3.3. Characterization of the Physical and Chemical Properties of the Antioxidant-HP-β-CD Complex

#### 3.3.1. Thermogravimetric Analysis

The abundance of flavonoids, easily recoverable as side products of pectin preparation from *citrus lemon* waste, prompted us to develop a coating procedure aimed at improving and preserving their biological activities along with increasing the solubility of phenolic compounds and their release.

For this reason, HP-β-CD was used as a complexing agent, and different ratios of antioxidant fraction were tested to find the best conditions for enhancing its stability and release over time. At first, to analyze the physical and chemical properties of the composite material, thermogravimetric analysis (TGA) was performed. For HP-β-CD inclusion complexes, the thermal stability of the guest molecules shifts to a higher temperature upon complexation due to host–guest interactions [28].

**Figure 3:**
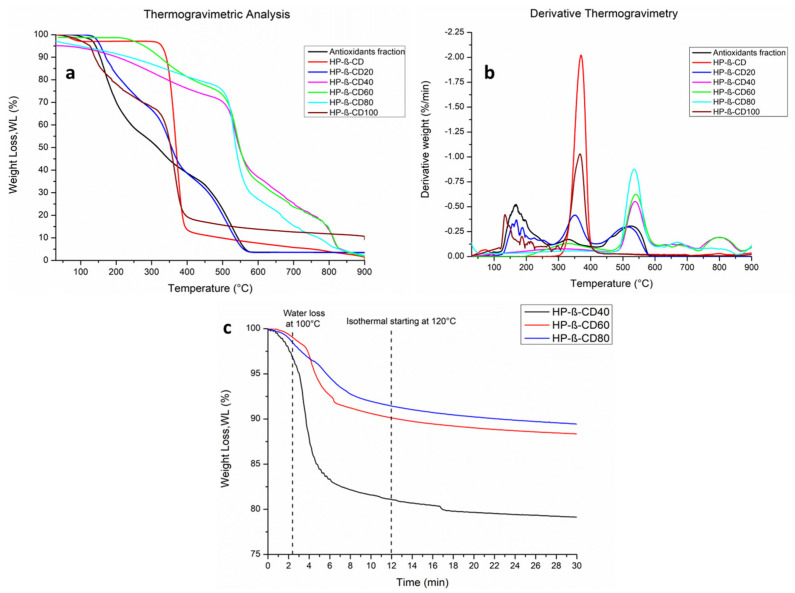
TGA, (**a**), and DTG, (**b**), of all samples; isothermal thermogravimetric analysis of HP-β-CD40, 60 and 80 (**c**).

In Figure 3, panels a and b, the TGA and DTG thermograms of the different inclusion complexes are reported. All the curves have been normalized with respect to the initial sample weights. TGA thermograms show that, generally, the complexes are less thermally stable than pure HP-β-CD. In particular, the lowest onset temperature (120 °C) is observed for the antioxidants fraction (black curve). Figure 3a shows that the thermal weight loss process of pure HP-β-CD occurs in a single stage in a range between 300 °C and 400 °C; in particular, in the DTG curve of HP- β -CD (Figure 3b), the temperature of maximum degradation rate (Tpeak) corresponds to 370 °C [29]. In this case, it is possible to observe that the thermogravimetric curve of the complex between the antioxidant fraction and HP-β-CD20 (Figure 3a) shows principally three thermal steps: the first thermal event, which is highlighted in all the curves, occurs for all the samples between 130–250 °C; this initial weight loss is associated with the thermal degradation of the bioactive molecules (~50–60%); the second thermal step centered between 300–400 °C, shows a rapid mass loss associated with the decomposition of HP-β-CD. Finally, the third step, evidenced as a very small and wide flex point at a temperature around 450–550 °C, corresponds to the thermal degradation of hydroxybenzene rings of the aromatic bioactive molecules [30]. From the thermogram analysis of HP-β-CD40, HP-β-CD60, and HP-β-CD80 complexes (Figure 3a), it is worthy to note that, by increasing the HP-β-CD content, the onset temperature of thermal degradation increases (TGA, Figure 3a) and the degradative kinetics slows in all the complexes, as evidenced by the shift of maximum degradation peaks (Tpeak) towards higher temperatures (DTG, Figure 3b). Evaluation of TGA and DTG curves also indicates that HP-β-CD inclusion complexes delay the weight loss of bioactive molecules during heating. Therefore, the thermal stability of antioxidants is improved when they are included in HP-β-CD. In addition, in Figure 3a, it is possible to note different starting degradation temperatures for the different complexes; in particular, the TGA curves relative to samples HP-β-CD40, and HP-β-CD80, show a minimal weight loss % up to 200 °C, while HP-β-CD60 keeps a constant weight up to 213 °C. Based on these results, to gain more information on the thermal stability of the complexes, they were subjected to isothermal TGA at 120 °C for 30 min, conditions that mimic autoclave sterilization. As it is possible to note in Figure 3c, HP-β-CD40 shows a significant initial weight loss %, while HP-β-CD60 and HP-β-CD80 have more similar behavior to each other and a less relevant weight loss %. Due to their thermal stability following the “autoclave mimicking” treatment, the latter complexes were chosen to be included in the subsequent pectin film preparation.

#### 3.3.2. Fourier-Transform Infrared Spectroscopy (FTIR)

**Figure 4:**
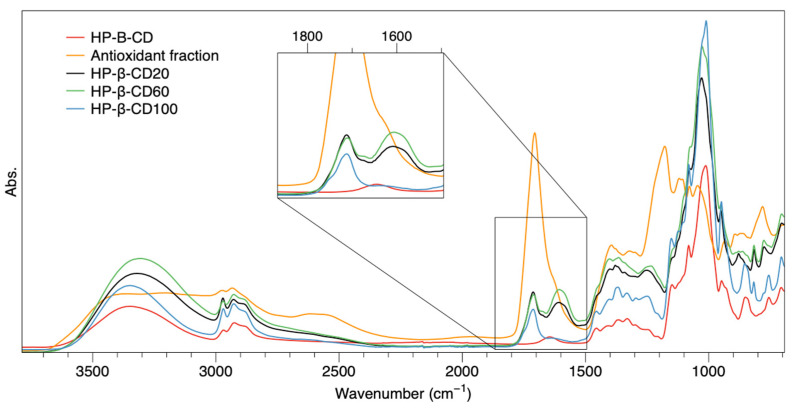
FTIR spectra of antioxidant fraction (orange), HP-β-CD (red), and inclusion complexes HP-β-CD20, HP-β-CD60, and HP-β-CD100 (black, green, and blue, respectively).

FTIR offers rapid analysis and needs little or no sample preparation. Moreover, it can offer information on different aspects simultaneously. This technique, in fact, was employed to identify the main functional groups and to assess inclusion complexes formation in the powdered state. The spectra of the antioxidant fraction, HP-β-CD, and the most representative inclusion complexes (HP-β-CD20, HP-β-CD60, and HP-β-CD100) are reported in Figure 4. The main characteristic vibrational modes of the antioxidant fraction can be assigned as follows: a broad band at 3600–3050 cm^−1^ is related to O–H stretching vibration; a band at 3000–2800 cm^−1^ is attributed to C–H stretching vibration of methyl and methylene groups. The carbonyl C=O stretching of the carboxyl group COO was indicated by the peak at 1705 cm^−1^, while the shoulder around 1600 cm^−1^ is ascribed to the asymmetric stretch vibration C=O of the free carboxyl. Region 1400–1200 cm^−1^ corresponds to C=C vibrations of the aromatic rings. The large band in the region 1200–1033 cm^−1^ was attributed to the C–O stretching vibration.

The spectrum of HP-β-CD shows an intense broad absorption band at 3500–3000 cm^−1^, which corresponds to O–H vibrations. The –CH_3_ and –CH_2_– vibrations were found around 2980 cm^−1^; on the other hand, the peak observed at 1657 cm^−1^ was associated with the H–O–H deformation band of the adsorbed water present in the HP-β-CD cavity [31]. Moreover, the large band observed in the range 1000–1200 cm^−1^ was attributed to C–O–C groups. Regarding the inclusion complexes, the first evidence of their formation can be correlated to the disappearance of signals related to H–O–H deformation, particularly noticeable in the sample HP-β-CD100. Moreover, most differences were found in the range 1700–1500 cm^−1^, which can also be ascribed to the formation of inclusion complexes, as suggested by Ji-Sang Kim [32] that attribute displacement, reduction, and disappearance of peaks in this range to the formation of complexes between flavonoids and HP-β-CD.

### 3.4. Assessment of the Antioxidant Properties

**Figure 5:**
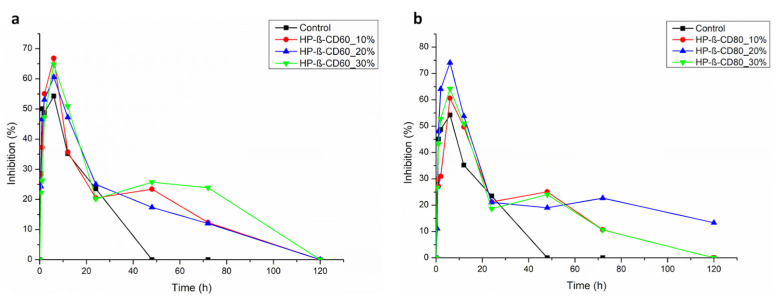
Time course of antioxidant activity of the films composed by (**a**) HP-β-CD60, (**b**) HP-β-CD80, and pectin.

Based on the thermal stability results, the HP-β-CD60 and HP-β-CD80 complexes were selected for the creation of pectin films containing the antioxidant mixtures complexed with HP-β-CD. Different amounts of the complexes were combined with pectin (as described in Section 2.8) loaded in a 96 multiwell plate, and the antioxidant activity measured up to 120 h, calculating % of DPPH inhibition (Figure 5a for HP-β-CD60 and Figure 5b for HP-β-CD80); in order to avoid the light damaging of antioxidant molecules, the samples were incubated in the dark at room temperature for the desired time.

Interestingly, the time course analysis showed that in all the samples analyzed, the scavenging activity after 10 h is always higher than the control; also, the complete loss of antioxidant activity, which is measured after 48 h in the non-encapsulated fraction, is shifted to 120 h for all the complexes with the exception of HP-β-CD80 20% that still maintains a residual 13% of activity after this time. As shown in Figure 5, the sample HP-β-CD80 20% also has the highest scavenging activity.

These data indicate that the creation of pectin films containing antioxidant molecules stabilized by HP-β-CD80 has potential biotechnological application for a localized delivery of these molecules, which can be gradual and long-lasting.

## 4. Conclusions

In this manuscript, we have focused our attention on the recovery of pectin and antioxidant molecules from the *citrus lemon* waste. After the extraction of pectin, the antioxidant fraction was analyzed, and its components were identified. At this point, in order to increase the stability of these antioxidant molecules and achieve a sustained release over time, they were encapsulated n HP-β-CD to form an inclusion complex.

The use of HP-β-CD complexes improves the thermal stability of antioxidant molecules, preserving unchanged the antioxidant activity of bioactive compounds. From these preliminary results, we selected the HP-β-CD60 and HP-β-CD80 samples in order to prepare pectin films with different amounts of antioxidants extracted from *citrus lemon* waste. The choice of the aforementioned complexes is due to their enhanced thermal stability, even in autoclave-like conditions, as a suitable sterilization protocol. Compared with the control film, the encapsulation form presented a more gradual release of antioxidants over time by the DPPH assay. On the basis of the results, the HP-β-CD80 complex shows the highest capacity of antioxidants inclusion, thermally stabilizing the bioactive molecules and promoting the longest-lasting scavenging activity. Future goals will evaluate, on the one hand, the possibility of these inclusion complexes as additives in thermoplastic polymer for food-packaging applications, in order to increase the shelf life of the food products, and on the other hand, the potential of these complexes as controlled release systems of bioactive molecules.

## Figures and Tables

**Table 1:** Validation methods by UHPLC.

Compounds	Regression Equation	R^2^	LOD mg/kg	LOQ mg/kg
***p*-Coumaric acid**	y = 114.17x + 35.47	0.999	0.081	0.315
**Ferulic acid**	y = 127.18x − 72.81	0.999	0.077	0.568
**Rutin**	y = 46.07x − 14.44	0.999	0.097	0.321
**Eriocitrin**	y = 41.392x − 38.81	0.999	0.357	19.082
**Narirutin**	y = 88.81x + 84.18	0.999	0.081	0.765
**Naringin**	y = 42.17x − 20.75	0.999	0.068	0.577
**Hesperidin**	y = 54.81x + 24.38	0.999	0.077	23.452

**Table 2:** Composition of the antioxidant/HP-β-CD complexes.

Identification Name	Antioxidants (mg)	HP-β-CD (mg)	Molar Ratio Antioxidants/ HP-β-CD
HP-β-CD20	100.0	52.4	1:0.4
HP-β-CD40	100.0	104.8	1:0.8
HP-β-CD60	100.0	157.2	1:1.2
HP-β-CD80	100.0	209.6	1:1.6
HP-β-CD100	100.0	250.9	1:2

**Table 3:** Antioxidant characterization of the extract analyzed by UHPLC (mg/kg).

Antioxidant Molecules	Amount
p-cumaric acid	6.06 ± 0.74
Ferulic acid	8.22 ± 2.33
Rutin	2.99 ± 0.65
Eriocitrin	167.10 ± 5.65
Narirutin	12.47 ± 2.95
Naringin	5.17 ± 0.42
Hesperidin	74.58 ± 2.19

## Data Availability

The datasets used and/or analyzed during the current study are available from the corresponding author on reasonable request.
